# The Fractality of Polar and Reed–Muller Codes †

**DOI:** 10.3390/e20010070

**Published:** 2018-01-17

**Authors:** Bernhard C. Geiger

**Affiliations:** Signal Processing and Speech Communication Laboratory, Graz University of Technology, 8010 Graz, Austria; geiger@ieee.org; Tel.: +43-316-873-4367

**Keywords:** polar codes, Reed–Muller codes, fractals, self-similarity

## Abstract

The generator matrices of polar codes and Reed–Muller codes are submatrices of the Kronecker product of a lower-triangular binary square matrix. For polar codes, the submatrix is generated by selecting rows according to their Bhattacharyya parameter, which is related to the error probability of sequential decoding. For Reed–Muller codes, the submatrix is generated by selecting rows according to their Hamming weight. In this work, we investigate the properties of the index sets selecting those rows, in the limit as the blocklength tends to infinity. We compute the Lebesgue measure and the Hausdorff dimension of these sets. We furthermore show that these sets are finely structured and self-similar in a well-defined sense, i.e., they have properties that are common to fractals.

## 1. Introduction

In his book on fractal geometry, Falconer characterizes a set F as a fractal if it has some of the following properties [1] (p. xxviii):

- F has a fine structure, i.e., there is detail on arbitrarily small scales
- F does not admit a description in traditional geometrical language, neither locally nor globally; it is irregular in some sense
- F has some form of self-similarity, at least approximate or statistical
- The fractal dimension of F exceeds its topological dimension
- F is defined in a simple, often recursive way

In this work, we investigate whether polar codes and Reed–Muller are fractal in above sense. For a blocklength of $2^{n}$, these codes are based on the *n*-fold Kronecker product $G\left(n\right):=F^{⊗n}$, where

(1) $$F:=\left[\begin{array}{cc} 1 & 0\\ 1 & 1 \end{array}\right]$$

i.e., on a simple, recursive operation. Based on this, it has long been suspected that Kronecker product-based codes possess a fractal nature. For example, the authors of [2] observed that G(n), when converted to a picture, resembles the Sierpinski triangle. In a personal communication [3], Abbe expressed his suspicion that the set of “good” polarized channels is fractal. Nevertheless, to the best of the author’s knowledge, a definite statement regarding this fractal nature has not been presented yet.

A rate-$K/2^{n}$ Kronecker product-based code is uniquely defined by a set F of *K* indices: Its generator matrix is the submatrix of G(n) consisting of the rows indexed by F. Letting F index the *K* rows of G(n) with the largest Hamming weight defines a Reed–Muller code. Alternatively, one can fix the *order* *r* of a Reed–Muller code, which defines F as the index set of all rows with a Hamming weight at least as large as *r* (see Section 4). For polar codes, the rows of G(n) can be interpreted as a communication channels. Then, a rate-$K/2^{n}$ polar code is defined by the set F indexing the *K* channels with the lowest Bhattacharyya parameters [4] (the “good” channels, see Section 2).

Although the sets F are important for the construction of polar and Reed–Muller codes, surprisingly little is known about their fractal properties. Recently, Renes, Sutter, and Hassani stated conditions under which the good (bad) channels derived from one binary-input memoryless channel are also good (bad) for another channel [5]. Moreover, the authors of [6,7] observed the self-similar structure of F by showing that polar and Reed–Muller codes are decreasing monomial codes.

In this paper, we analyze the fractal properties of F for polar codes (Section 3) and Reed–Muller codes (Section 5). In contrast to [6,7], we study the properties of F for infinite blocklengths, i.e., for n→∞. Specifically, we compute the Hausdorff dimension of F, show that it is self-similar, and that it has detail on arbitrarily small scales (e.g., F is symmetric and dense in some well-defined containing set). Each of these results is relatively easy to obtain once appropriate definitions have been put in place. Taken as a whole, however, they paint an interesting picture and make a convincing point for the claim that polar and Reed–Muller codes are fractal.

The presented results will improve our understanding of polar and Reed–Muller codes, even though we have to admit that their practical implication (e.g., in code construction) still eludes us. Nevertheless, our results may apply in areas beyond channel coding: Arıkan’s polarization technique was used to polarize Rényi information dimension [8] and to construct high-girth matrices [9]. Moreover, Nasser showed that a sufficient and necessary condition for a binary operation to be polarizing is that it is uniformity preserving and that its inverse is strongly ergodic [10,11]. We are convinced that fractality carries over to these applications as well and that an analysis similar to ours can deepen understanding.

Since we consider the case n→∞, the set F indexes a subset of $\Omega :={\left\{0,1\right\}}^{\infty }$, the set of infinite binary sequences. We let $b:=(b_{1}b_{2}\cdots )\in \Omega$ and abbreviate $b^{n}:=\left(b_{1}b_{2}\cdots b_{n}\right)$. Let $\bar{b}:=\left({\bar{b}}_{1}{\bar{b}}_{2}\cdots \right)$ where ${\bar{b}}_{i}:=1-b_{i}$. Let furthermore (Ω,A,P) be a probability space with A the Borel field generated by the cylinder sets $S\left(b^{n}\right):=\left\{w\in \Omega :\;w_{1}=b_{1},\cdots ,w_{n}=b_{n}\right\}$ and P a probability measure satisfying $P\left(S\left(b^{n}\right)\right)=1/2^{n}$. In the following, we represent every infinite binary sequence b∈Ω by a point in the unit interval [0,1]. The mapping between Ω and [0,1] is given by

(2) $$f\left(b\right):=\sum_{n=1}^{\infty }\frac{b_{n}}{2^{n}}.$$

**Lemma** **1** ([12] (Exercises 7–10, p. 80)). *Let $B_{\left[0,1\right]}$ be the Borel σ-algebra on [0,1] and let λ be the Lebesgue measure. Let furthermore $D:=\left[0,1\right]\cap \left\{p/2^{n}:\;p\in Z,n\in N\right\}$ denote the set of dyadic rationals in the unit interval. Then, the function $f:\;\left(\Omega ,A\right)\to (\left[0,1\right],B_{\left[0,1\right]})$ in* (2) *satisfies the following properties:*

*1.*  f is measurable
*2.*  f is bijective on $\Omega \backslash f^{-1}\left(D\right)$
*3.*  for all $I\in B_{\left[0,1\right]}$, $P(f^{-1}\left(I\right))=\lambda \left(I\right)$

**Example** **1.** *Lemma 1 states that f is not injective in general. The reason is that dyadic rationals have a non-unique binary expansion. For example, f maps both b=(01111111⋯) and b=(10000000⋯) to 0.5, where we call the latter binary expansion terminating.*

## 2. Preliminaries for Polar Codes

Let W:{0,1}→Y be a binary-input memoryless channel with finite output alphabet Y, (symmetric) capacity 0≤I(W)≤1, and with Bhattacharyya parameter

(3) $$Z\left(W\right):=\sum_{y\in Y}\sqrt{W(y|0)W(y|1)}.$$

It can be shown using [13] (Proposition 1) that Z(W)=0⇔I(W)=1 and Z(W)=1⇔I(W)=0. We say that the channel *W* is *symmetric* if there exists a permutation π:Y→Y such that $\pi^{-1}=\pi$ and, for every y∈Y, W(y|0)=W(π(y)|1).

Arıkan’s polarization technique [13] *combines and splits* two channel uses of *W* into one use of a “worse” channel

(4) $$W_{2}^{0}\left(y_{1}^{2}|u_{1}\right):=\frac{1}{2}\sum_{u_{2}}W\left(y_{1}|u_{1}⊕u_{2}\right)W\left(y_{2}|u_{2}\right)$$

and one use of a “better” channel

(5) $$W_{2}^{1}\left(y_{1}^{2},u_{1}|u_{2}\right):=\frac{1}{2}W\left(y_{1}|u_{1}⊕u_{2}\right)W\left(y_{2}|u_{2}\right)$$

where $u_{1},u_{2}\in \left\{0,1\right\}$ and $y_{1},y_{2}\in Y$. The combining operation encodes two input bits by *F* in (1); transmitting them via two channel uses of *W* creates a vector channel. This vector channel can then be split into the two virtual binary-input memoryless channels indicated in (4) and (5). The better (worse) channel obtained by polarization has a larger (smaller) capacity than the original channel *W*, i.e., $I\left(W_{2}^{0}\right)\leq I\left(W\right)\leq I\left(W_{2}^{1}\right)$—the inequalities are strict if 0<I(W)<1. The sum capacity equals two times the capacity of the original channel, i.e., $I\left(W_{2}^{0}\right)+I\left(W_{2}^{1}\right)=2I\left(W\right)$ [13] (Proposition 4). Similarly, polarization has an effect on the Bhattacharyya parameter:

**Lemma** **2** (Bounds on the Bhattacharyya Parameter). (6) $$\begin{array}{cc} Z(W_{2}^{1}) & =g_{1}\left(Z\left(W\right)\right):=Z^{2}\left(W\right)\leq Z\left(W\right) \end{array}$$

(7) $$\begin{array}{cc} Z\left(W\right)\leq Z\left(W\right)\sqrt{2-Z^{2}\left(W\right)}=:h_{0}\left(Z\left(W\right)\right)\leq Z\left(W_{2}^{0}\right) & \overset{\left(a\right)}{\leq }g_{0}\left(Z\left(W\right)\right):=2Z\left(W\right)-Z^{2}\left(W\right) \end{array}$$

*with equality in (a) if W is a binary erasure channel (BEC).*

**Proof.**  The equality and inequality in (6) follow from [13] (Proposition 7) and the fact that Z(W)≤1, respectively. The inequalities in (7) follow from the fact that Z(W)≤1, from [14] (Lemma 20), and from [13] (Proposition 7). The last inequality becomes an equality if *W* is a BEC [13] (Proposition 7). ☐

For larger blocklengths $2^{n}$, n>1, we apply the polarization procedure recursively and obtain, for $b^{n}\in {\left\{0,1\right\}}^{n}$,

(8) $$\left(W_{2^{n}}^{b^{n}},W_{2^{n}}^{b^{n}}\right)\to \left(W_{2^{n+1}}^{b^{n}0},W_{2^{n+1}}^{b^{n}1}\right)$$

where $b^{n}0$ and $b^{n}1$ denote the sequences of zeros and ones obtained by appending 0 and 1 to $b^{n}$, respectively. Note that the functions $g_{1}$, $g_{0}$, and $h_{0}$ from Lemma 2 are non-negative and non-decreasing and map the unit interval onto itself. Hence, the inequalities in (7) are preserved under composition:

(9) $$\begin{array}{cc} Z(W_{2^{n}}^{b^{n}}) & \leq p_{b^{n}}\left(Z\left(W\right)\right):=g_{b_{n}}\left(g_{b_{n-1}}\left(\cdots g_{b_{1}}\left(Z\left(W\right)\right)\cdots \right)\right) \end{array}$$

(10) $$\begin{array}{cc} Z(W_{2^{n}}^{b^{n}}) & \geq q_{b^{n}}\left(Z\left(W\right)\right):=h_{b_{n}}\left(h_{b_{n-1}}\left(\cdots h_{b_{1}}\left(Z\left(W\right)\right)\cdots \right)\right) \end{array}$$

where $h_{1}\equiv g_{1}$.

Applying this recursive polarization infinitely often leads to a situation in which almost all channels are either perfect or useless, i.e., either $I(W_{\infty }^{b})=1$ or $I(W_{\infty }^{b})=0$ for $b\in {\left\{0,1\right\}}^{\infty }$. This is the assertion of Arıkan’s polarization theorem:

**Proposition** **1** ([13] (Proposition 10)). *With probability one, the limit RV $I_{\infty }\left(b\right):=I\left(W_{\infty }^{b}\right)$ takes values in the set {0,1}: $P\left(I_{\infty }=1\right)=I\left(W\right)$ and $P\left(I_{\infty }=0\right)=1-I\left(W\right)$.*

If we stop the polarization procedure at a finite blocklength $2^{n}$ for *n* large enough, then still most of the resulting $2^{n}$ channels are either almost perfect or almost useless (i.e., the channel capacities are close to one or to zero). The idea of polar coding is to transmit data only on those channels that are almost perfect. The generator matrix of a blocklength-$2^{n}$ polar code is thus the submatrix of G(n) consisting of rows indexed by F, where F contains the indices corresponding to the *K* virtual channels with the largest capacities. Determining this set F is inherently difficult, since (whenever *W* is not a BEC) the cardinality of the output alphabet increases exponentially in $2^{n}$ [15] (Chapter 3.3), [16] (p. 36). Tal and Vardy proposed an approximate construction method based on reducing the output alphabet and showing that the resulting channels are either upgraded or degraded w.r.t. the channel of interest [17] (see also Korada’s PhD thesis [16] (Definition 1.7 & Lemma 1.8)). These upgrading/degrading properties are important tools in our proofs.

**Definition** **1** (Channel Upgrading and Degrading). *A channel $W^{-}:\;\left\{0,1\right\}\to Z$ is* degraded *w.r.t. the channel W (short: $W^{-}≼W$) if there exists a channel Q:Y→Z such that*

(11) $$W^{-}\left(z|u\right)=\sum_{y\in Y}W\left(y|u\right)Q\left(z|y\right).$$

*A channel $W^{+}:\;\left\{0,1\right\}\to Z$ is* upgraded *w.r.t. the channel W (short: $W^{+}≽W$) if there exists a channel P:Z→Y such that*

(12) $$W\left(y|u\right)=\sum_{z\in Z}W^{+}\left(z|u\right)P\left(y|z\right).$$

Moreover, $W^{+}≽W$ if and only if $W≼W^{+}$.

Upgraded (degraded) channels remains upgraded (degraded) during polarization:

**Lemma** **3** ([16] (Lemma 4.7) & [17] (Lemmas 3 & 5)). *Suppose that $W^{-}≼W≼W^{+}$. Then,*

(13) $$\begin{array}{ccccc} I(W^{-}) & \leq & I(W) & \leq & I(W^{+})\\ Z(W^{-}) & \geq & Z(W) & \geq & Z(W^{+})\\ {\left(W^{-}\right)}_{2}^{1} & ≼ & W_{2}^{1} & ≼ & {\left(W^{+}\right)}_{2}^{1}\\ {\left(W^{-}\right)}_{2}^{0} & ≼ & W_{2}^{0} & ≼ & {\left(W^{+}\right)}_{2}^{0}. \end{array}$$

**Lemma** **4** ([15] (p. 9) & [6] (Lemma 3)). *$W≼W_{2}^{1}$. If W is symmetric, then $W_{2}^{0}≼W≼W_{2}^{1}$.*

**Proof.**  By choosing

(14) $$P\left(y|y_{1}^{2},u_{1}\right)=\left\{\begin{array}{cc} 1, & \mathrm{if}\;y=y_{2}\\ 0, & \mathrm{else}. \end{array}\right.$$

one can show that $W≼W_{2}^{1}$. To show that also $W_{2}^{0}≼W$ for symmetric channels, take [6] (Lemma 3)

(15) $$Q\left(y_{1}^{2}|y\right)=\left\{\begin{array}{cc} \frac{1}{2}W\left(y_{2}|0\right) & \;\mathrm{if}\;y_{1}=y\\ \frac{1}{2}W\left(y_{2}|1\right) & \;\mathrm{if}\;y_{1}=\pi \left(y\right)\\ 0 & \;\mathrm{else}. \end{array}\right.$$

 ☐

## 3. Fractal Properties of the Sets of Good and Bad Channels

We next investigate the behavior of the set F as we let the blocklength tend to infinity, i.e., as n→∞. This set indexes all sequences *b* for which we obtain $I(W_{\infty }^{b})=1$. With the help of (2), we map these sequences to a subset of the unit interval, which we will call the set of *good channels*.

**Definition** **2** (The Good and the Bad Channels). *Let G denote the set of good channels, i.e.,*

(16) $$x\in G\Leftrightarrow \exists b\in f^{-1}\left(x\right):\;I\left(W_{\infty }^{b}\right)=1.$$

*Let B denote the set of bad channels, i.e.,*

(17) $$x\in B\Leftrightarrow \exists b\in f^{-1}\left(x\right):\;I\left(W_{\infty }^{b}\right)=0.$$

If I(W)=0, then all polarized channels are useless and we have B=[0,1]. Similarly, if I(W)=1, then all polarized channels are perfect and we have G=[0,1]. We hence assume throughout this section that the channel *W* is nontrivial, i.e., that 0<I(W)<1.

**Proposition** **2** (Denseness). *G∩B=D, i.e., the good and bad channels are dense in the unit interval. Moreover, G\D and B\D are dense in [0,1].*

**Proof.**  See Appendix A. ☐

It is not really surprising that G and B are not disjoint; this is a direct consequence of the fact that *f* is not injective. It is not obvious, however, that the intersection exhausts the set on which *f* is non-injective. A consequence of this proposition is that there is no interval that contains only good channels. This has implications for code construction techniques. Indeed, the authors of [18,19] suggest that, for a polar code of a given blocklength, one may stop polarizing channels at shorter blocklengths and use copies of these channels rather than their polarization. For example, they suggest to use the channels $\left(W_{2^{n}}^{b^{n}},W_{2^{n}}^{b^{n}}\right)$ rather than the channels $\left(W_{2^{n+1}}^{b^{n}0},W_{2^{n+1}}^{b^{n}1}\right)$ if $I(W_{2^{n}}^{b^{n}})$ is sufficiently large. Such a procedure can be justified if further polarizing $W_{2^{n}}^{b^{n}}$ to the desired blocklength will lead to including all channels polarized from $W_{2^{n}}^{b^{n}}$ in the code. Such a justification can never appear for polar codes with unbounded blocklength: Stopping polarizing at a given blocklength $2^{n}$ for a given polarization sequence and using copies of the resulting channel $W_{2^{n}}^{b^{n}}$ is equivalent to including a dyadic interval in the index set. This dyadic interval contains, by Proposition 2, bad channels, which shows that this choice is suboptimal.

**Proposition** **3** (Symmetry). *There exists a function ϑ, defined for almost all values in [0,1], that is independent of W and satisfies 0≤ϑ(x)≤1 and ϑ(1−x)=1−ϑ(x). Let x∈[0,1] be such that ϑ(x) is defined. Then, ϑ(x)>Z(W) implies x∈G. If W is a BEC, then ϑ(x)<Z(W) implies x∈B.*

**Proof.**  See Appendix B. ☐

Proposition 3 has two implications. The first implication concerns the *alignment* of the sets G and $G^{\prime }$ for two different channels *W* and $W^{\prime }$. Specifically, it is connected to the question whether $Z\left(W\right)\geq Z\left(W^{\prime }\right)$ implies $G\subseteq G^{\prime }$. In general, the answer is negative [5]. Indeed, it may happen that for some b∈Ω, we have $I(W_{\infty }^{b})=1$ despite Z(W)>ϑ(f(b)), i.e., that the polarized channel turns out to be good even though the sufficient condition from Proposition 3 is not fulfilled. Such a situation cannot occur for BECs, as Proposition 3 shows. Hence, the set of good channels for a BEC is also good for any binary-input memoryless channel with a smaller Bhattacharyya parameter [20].

**Figure 1:**
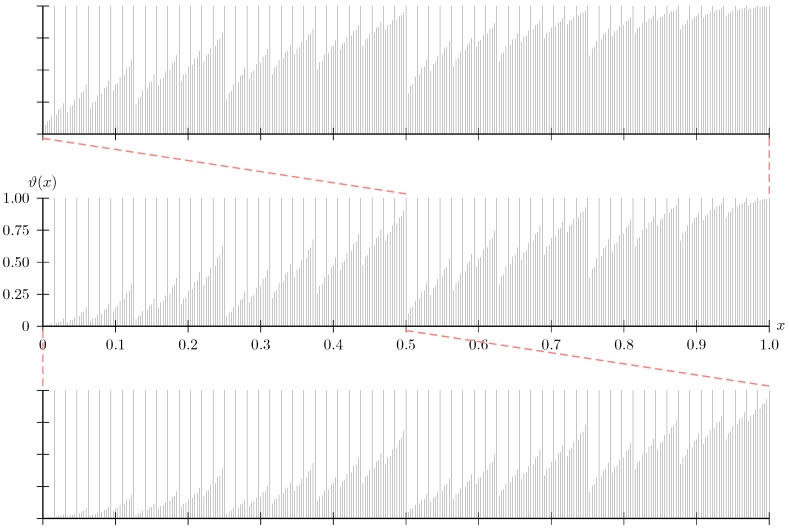
The polar fractal. The center plot shows the thresholds ϑ(x) for a finite set of values x∈[0,1]; the bottom and the top plots show thresholds for equally many values in the sets [0,0.5] and [0.5,1], respectively. One can observe how the thresholds are ordered, i.e., thresholds in the top plot exceed those in the center plot, which exceed those in the bottom plot. For a binary erasure channel (BEC) *W*, the indicator function of G is obtained by setting each value in the plot to one (zero) if the Bhattacharyya parameter Z(W) is smaller (larger) than the threshold. Note that this plot illustrates the behavior of G in the limit n→∞. Note further that the figure illustrates the symmetry of ϑ(x) claimed in Proposition 3. The MATLAB code to generate these thresholds is available as Supplementary Material.

The second implication is that, at least for BECs, the sets G and B are symmetric. Indeed, if ϑ(x)≠Z(W), then x∈G implies 1−x∈B. This symmetry is visible in the polar fractal that we display in Figure 1.

It is possible to define ϑ for x∈D. We know from Proposition 2 that dyadic rationals are both good and bad, hence setting ϑ(x)=1 for every x∈D leads to D⊆G. (The fact that also D⊆B is not captured by nor in conflict with this setting.) The question whether the function ϑ can be defined for x∈Q\D is more interesting. In this case, the binary expansion is unique and *recurring*, i.e., there is a length-*k* sequence $a^{k}\in {\left\{0,1\right\}}^{k}$ such that $f(b^{n}a^{k}a^{k}a^{k}\cdots )=x$ for some $b^{n}\in {\left\{0,1\right\}}^{n}$. It is straightforward to show that for every non-trivial sequence $a_{k}$ (i.e., $a_{k}$ contains zeros and ones), $p_{a^{k}}$ is from [0,1] to [0,1], non-negative, and non-decreasing with vanishing derivatives at 0 and 1. Since this ensures that $p_{a^{k}}\left(z\right)<z$ for *z* close to zero and $p_{a^{k}}\left(z\right)>z$ for *z* close to one, the operation $z_{i+1}=p_{a^{k}}\left(z_{i}\right)$ constitutes an iterated function system with attracting fixed points at z=0 and z=1. Note further that, since $p_{a^{k}}$ corresponds to the recurring part of the binary expansion of *x*, $Z(W_{\infty }^{b^{n}a^{k}a^{k}\cdots })$ will be bounded from above by the value to which this iterated function system converges after being initialized with $Z(W_{2^{n}}^{b^{n}})$. To show that Proposition 3 holds for x∈Q\D requires showing that $p_{a^{k}}$ intersects the identity function only once on (0,1), i.e., that there is no attracting fixed point on this open interval. We leave this problem for future investigation.

**Example** **2.** Let x=2/3, hence $f^{-1}\left(x\right)=101010101\cdots$. We determine the fixed points of the iterated function system corresponding to one period of the recurring sequence, i.e, the fixed points of $p_{10}\left(z\right)=2z^{2}-z^{4}$. These are given by the roots of $p_{10}\left(z\right)-z$, which are z=0, z=1, and $z=(\pm \sqrt{5}-1)/2$. One of these latter nontrivial roots lies outside [0,1] and is hence irrelevant. The remaining root determines the threshold, $\vartheta \left(2/3\right)=\left(\sqrt{5}-1\right)/2$.

*Now suppose that W is a BEC with Bhattacharyya parameter Z(W)=ϑ(2/3). Since ϑ(2/3) is a fixed point, we get $Z\left(W_{\infty }^{f^{-1}\left(2/3\right)}\right)=Z\left(W\right)\notin \left\{0,1\right\}$. This illustrates that Proposition 1 holds only almost surely.*

**Proposition 4** (Lebesgue Measure & Hausdorff Dimension). *G is a Borel set and has Lebesgue measure λ(G)=I(W). B is a Borel set and has Lebesgue measure λ(B)=1−I(W). Therefore, the Hausdorff dimensions of G and B satisfy d(G)=d(B)=1.*

**Proof.**  See Appendix C. ☐

Loosely speaking, the Lebesgue measure of G is the asymptotic equivalent of the rate of the “infinite-blocklength” polar code for the channel *W*. The fact that λ(G)=I(W) states that the rate approaches the symmetric capacity of *W*. A positive Lebesgue measure and a Hausdorff dimension equal to one are not indicators of fractality.

The last fractal property we consider is self-similarity. As Falconer notes [1] (p. xxviii), self-similarity often occurs only approximately. What we show in the following proposition is that the set G is *quasi self-similar*. Along the same lines, the quasi self-similarity of B can be shown.

**Proposition** **5** (Self-Similarity). *Let $G_{n}\left(k\right):=G\cap \left[\left(k-1\right)2^{-n},k2^{-n}\right]$ for $k=1,\ldots ,2^{n}$. $G=G_{0}\left(1\right)$ is* quasi self-similar *in the sense that, for all n and all k, $G_{n}\left(k\right)=G_{n+1}\left(2k-1\right)\cup G_{n+1}\left(2k\right)$ is quasi self-similar to its right half:*

(18) $$G_{n}\left(k\right)\subseteq 2G_{n+1}\left(2k\right)-k2^{-n}$$

*If W is symmetric, $G_{n}\left(k\right)$ is quasi self-similar:*

(19) $$2G_{n+1}\left(2k-1\right)-\left(k-1\right)2^{-n}\subseteq G_{n}\left(k\right)\subseteq 2G_{n+1}\left(2k\right)-k2^{-n}$$

**Proof.**  See Appendix D. ☐

In other words, at least for a symmetric channel, G is composed of two similar copies of itself (see Figure 1). The self-similarity is closely related to the fact that polar codes are decreasing monomial codes [6] (Theorem 1).

**Example** **3.** *We want to determine whether 1/3∈G for a given BEC W. This question translates the questions whether $1/6\in G_{1}\left(1\right)$ and whether $2/3\in G_{1}\left(2\right)$. Along the lines of Example 2, we obtain ϑ(1/6)≈0.214, ϑ(1/3)≈0.382, and ϑ(2/3)≈0.618, i.e., ϑ(1/6)<ϑ(1/3)<ϑ(2/3). Since W is a BEC, we can connect this with Proposition 3 and thus obtain the inclusion indicated in Proposition 5.*

## 4. Preliminaries for Reed–Muller Codes

An order-*r*, length-$2^{n}$ Reed–Muller code is defined by having a generator matrix $G_{RM}\left(r,n\right)$ composed of all length-$2^{n}$ sequences with a Hamming weight larger than $2^{n-r}$. For example, we have $G_{RM}\left(n,n\right)=G\left(n\right)$, while $G_{RM}\left(0,n\right)$ is a single row vector containing only ones (length-$2^{n}$ repetition code). To make this more precise, let $w\left(b^{n}\right)=\sum_{i=1}^{n}b_{i}$ be the *Hamming weight* of $b^{n}\in {\left\{0,1\right\}}^{n}$ and let $s_{i}\left(n\right)$ be the *i*-th row of G(n). Then, the generator matrix $G_{RM}\left(r,n\right)$ of an order-*r*, length-$2^{n}$ Reed–Muller code consists of the rows of G(n) indexed by [4]

(20) $$F=\{i\in \left\{1,\ldots ,2^{n}\right\}:\;w\left(s_{i}\left(n\right)\right)\geq 2^{n-r}\}.$$

To analyze the effect of doubling the block length, note that

(21) $$G\left(n+1\right):=\left[\begin{array}{cc} G(n) & 0\\ G(n) & G(n) \end{array}\right].$$

Assume that we indicate the rows of G(n) by a sequence of binary numbers, i.e., let the *i*-th row be indexed by $h_{n}\left(b^{n}\right):=2^{n}\sum_{l=1}^{n}b_{l}2^{-l}$. Furthermore, let $0b^{n}$ and $1b^{n}$ denote the sequences of zeros and ones obtained by prepending 0 and 1 to $b^{n}$, respectively. Clearly, $h_{n+1}\left(0b^{n}\right)=h_{n}\left(b^{n}\right)$ and $h_{n+1}\left(1b^{n}\right)=h_{n}\left(b^{n}\right)+2^{n}$. Combining this with (21) yields

$$\begin{array}{cc} w(s_{h_{n+1}\left(0b^{n}\right)}\left(n+1\right)) & =w(s_{h_{n}\left(b^{n}\right)}\left(n\right))\\ w(s_{h_{n+1}\left(1b^{n}\right)}\left(n+1\right)) & =2w(s_{h_{n}\left(b^{n}\right)}\left(n\right)). \end{array}$$

Defining G(0):=1, we thus get

(22) $$w\left(s_{h_{n}\left(b^{n}\right)}\left(n\right)\right)=2^{w(b^{n})}$$

and

(23) $$F=h_{n}\left(\{b^{n}\in {\left\{0,1\right\}}^{n}:\;2^{w(b^{n})}\geq 2^{n-r}\}\right).$$

In Section 5, we will analyze the properties of F in the limit as *n* tends to infinity. An important ingredient in our proofs is the concept of *normal numbers*.

**Definition** **3** (Normal Numbers). *A number x∈[0,1] is called* simply normal to base 2*(x∈N) if and only if*

(24) $$\exists b\in f^{-1}\left(x\right):\;\lim_{n\to \infty }\frac{w(b^{n})}{n}=\frac{1}{2}.$$

In general, a number is simply normal to base *M* if the fraction of its digits used in its *M*-ary expansion is 1/M. A number is called normal if this property not only holds for digits, but also for subsequences: a number is normal in base *M* if, for each k≥1, the fraction of each length-*k* sequences used in its *M*-ary expansion is $1/M^{k}$. It immediately follows that a normal number is simply normal. The converse is in general not true:

**Example** **4.** *Let x=1/3, hence b=010101⋯. x is simply normal to base 2, but not normal (since the sequences 00 and 11 never occur). Let x=1/7, hence b=001001001⋯. x is neither normal nor simply normal. Let x∈D, hence b is either terminating ($\lim_{n\to \infty }w\left(b^{n}\right)/n=0$) or non-terminating ($\lim_{n\to \infty }w\left(b^{n}\right)/n=1$). Dyadic rationals are not simply normal.*

**Lemma** **5** (Borel’s Law of Large Numbers, cf. [21] (Corollary 8.1, p. 70)). *Almost all numbers in [0,1] are simply normal, i.e.,*

(25) λ(N)=1.

Despite this result, there are uncountably many numbers in the unit interval which are not normal. Moreover, the set of numbers that are not normal is *superfractal*, i.e., it has a Hausdorff dimension equal to one although it has zero Lebesgue measure [22].

## 5. Fractal Properties of the Set of Heavy Codewords

If we let *n* tend to infinity, the definition of F in (23) becomes problematic. Rather than looking at order-*r*, length-$2^{n}$ Reed–Muller codes, we investigate order-(1−ρ)n, length-$2^{n}$ codes, where we assume that ρn is integer. In other words, we assume that the threshold for the Hamming weight increases linearly with the blocklength. This gives rise to the definition of *heavy codewords*:

**Definition** **4** (The Heavy Codewords). *Let H(ρ) denote the set of ρ-heavy codewords, i.e.,*

(26) $$x\in H\left(\rho \right)\Leftrightarrow \exists b\in f^{-1}\left(x\right):\;\underset{n\to \infty }{\lim\;\inf}\frac{2^{w(b^{n})}}{2^{n\rho }}\geq 1.$$

Loosely speaking, the set of heavy codewords corresponds to those rows of G(n) that asymptotically have a *fractional* Hamming weight larger than a given threshold.

**Example** **5.** H(1)={1}. This follows from the fact that 1 is the only number in the unit interval with a binary expansion consisting only of ones. H(0)=[0,1]. This follows from the fact that $w(b^{n})\geq 0$.

**Proposition** **6** (Denseness). *For all ρ∈[0,1), D⊂H(ρ). Moreover, for ρ∈(0,1), H(ρ)\D and its complement are dense in [0,1].*

Similarly as for polar codes, also Reed–Muller codes are such that no interval is contained in either H(ρ) or its complement (unless in the trivial cases H(0) and H(1)). This is again in contrast with the intuition one obtains for Reed–Muller code with finite blocklength. Suppose we fix *n* to be even and set r=n/2, i.e., we require that at least one half of the bits in $b^{n}$ are one. The matrix G(n) resembles a Sierpinski triangle, as depicted in [2] (Figure 2). In our notation, the set F indexes none of the first $2^{n/2}-1$ rows of G(n), since they cannot have sufficient Hamming weight. Consequently, the transition as n→∞ creates complications that are not present for finite *n*, and one needs to depart from intuition based on these finite-blocklength considerations.

**Proof.**  See Appendix E. ☐

**Proposition 7** (Lebesgue Measure & Hausdorff Dimension). *H(ρ) is Lebesgue measurable and has Lebesgue measure*

(27) $$\lambda \left(H\left(\rho \right)\right)=\left\{\begin{array}{cc} 1, & \;\mathrm{if}\;\rho <1/2\\ 0, & \;\mathrm{if}\;\rho \geq 1/2. \end{array}\right.$$

*The Hausdorff dimension satisfies*

(28) $$d\left(H\left(\rho \right)\right)\left\{\begin{array}{cc} =1, & \;\mathrm{if}\;\rho \leq 1/2\\ \geq h_{2}\left(\rho \right), & \;\mathrm{if}\;\rho >1/2 \end{array}\right.$$

*where $h_{2}\left(x\right):=-x\log_{2}x-\left(1-x\right)\log_{2}\left(1-x\right)$.*

**Proof.**  See Appendix F. ☐

Loosely speaking, the Lebesgue measure of H(ρ) is the asymptotic equivalent of the rate of the fractional order-ρ Reed–Muller code. As we showed in Proposition 4, the Lebesgue measure of G is equal to the symmetric capacity of *W*. In contrast, the set H(ρ) does not depend on *W*. Rather, Proposition 7 suggests that the order parameter ρ induces a *phase transition* for the rate of Reed–Muller codes: If ρ<1/2, the “infinite-blocklength” Reed–Muller code consists of almost all (in the sense of Lebesgue measure) possible binary sequences. In contrast, if ρ≥1/2, the “infinite-blocklength” Reed–Muller code consists of almost no codewords (again, in the sense of Lebesgue measure).

Let us briefly consider the case ρ=1/2. For this case, Proposition 7 states that H(ρ) is a Lebesgue null set that has a Hausdorff dimension equal to 1. Thus, the set H(1/2) is a superfractal. Unfortunately, we were not able to give an exact expression for the Hausdorff dimension of H(ρ) for ρ>1/2. While the set of all non-normal numbers is superfractal, we are not sure if this holds also for the specific proper subset H(ρ).

The sets G and B exhibit self-similarity, i.e., detailed structure on every scale (cf. Figure 1). We next show that also H(ρ) is self-similar. At least for H(0) and H(1) (cf. Example 5) this is as trivial as the self-similarity of a point or a line. For ρ∈(0,1) this self-similarity is more interesting, and related to the fact that Reed–Muller codes are decreasing monomial codes [6] (Proposition 2).

**Proposition** **8** (Self-Similarity). *Let $H_{n}\left(\rho ,k\right):=H\left(\rho \right)\cap \left[\left(k-1\right)2^{-n},k2^{-n}\right]$ for $k=1,\ldots ,2^{n}$. $H\left(\rho \right)=H_{0}\left(\rho ,1\right)$ is* quasi self-similar *in the sense that, for all n and all k, $H_{n}\left(\rho ,k\right)=H_{n+1}\left(\rho ,2k-1\right)\cup H_{n+1}\left(\rho ,2k\right)$ is quasi self-similar:*

(29) $$2H_{n+1}\left(\rho ,2k-1\right)-\left(k-1\right)2^{-n}\subseteq H_{n}\left(\rho ,k\right)\subseteq 2H_{n+1}\left(\rho ,2k\right)-k2^{-n}.$$

**Proof.**  See Appendix G. ☐

## 6. Discussion and Outlook

That Kronecker product-based codes possess fractal properties has long been suspected. The present manuscript contains several results that back this suspicion with solid mathematical analyses. Specifically, we assumed that the blocklength tends to infinity and investigated the properties of the set G of virtual channels that are perfect and the set H(ρ) of codewords that have a fractional Hamming weight no less than ρ. Since both polar codes and Reed–Muller codes are obtained by a simple, recursive procedure, it remains to investigate whether the sets G and H(ρ) satisfy any of the following properties [1] (p. xxviii):

- The set has a fine structure, i.e., there is detail on arbitrarily small scales;
- It does not admit a description in traditional geometrical language, neither locally nor globally; it is irregular in some sense;
- It has some form of self-similarity, at least approximate or statistical;
- The fractal dimension of the set exceeds its topological dimension.

Indeed, the sets G and H(ρ) possess a fine structure in the sense that they are dense in the unit interval, but that also their complements are dense in the unit interval (cf. Propositions 2 and 6). Therefore, at an arbitrarily small scale, the sets G and H(ρ) admit no simple description in geometrical language. Both of these sets are self-similar in a specific sense, as we outlined in Propositions 5 and 8. Finally, while G has a fractal dimension of one (cf. Proposition 4), the set H(ρ) has, for a certain range of ρ, a positive (fractional?) Hausdorff dimension despite being a Lebesgue null set. This result, which we proved in Proposition 7, is one of the defining properties of a fractal set.

One reviewer pointed out that our definition of H(ρ) can be complemented by a different one. Specifically, while H(ρ) indexes the codewords with a fractional Hamming weight not smaller than ρ, one could define a set $H^{\prime }\left(R\right)$ indexing the codewords of a Reed–Muller code with rate *R*. In other words, while H(ρ) is parameterized via the *fractional order* of the code, $H^{\prime }\left(R\right)$ is parameterized via its rate. We expect that the Lebesgue measure of the (adequately defined) set $H^{\prime }\left(R\right)$ should be *R* and that, thus, its Hausdorff dimension equals one. An appropriate definition of $H^{\prime }\left(R\right)$ is tied to the set F of a rate-*R*, length-$2^{n}$ Reed–Muller code (such as is our Definition 4). Since finding such a definition has so far eluded us, we postpone this investigation to future work.

Another obvious extension of our work are non-binary polar and Reed–Muller codes. For example, consider an ℓ×ℓ matrix with entries from {0,…,q−1}, where *q* is prime. One can show that this matrix is polarizing as long as it is not upper-triangular [15] (Theorem 5.2). We believe that our analysis can be replicated by considering the *ℓ*-ary expansion of real numbers in [0,1]. Along the same lines, it would be interesting to examine the properties of *q*-ary Reed–Muller codes, e.g., [23,24].

## Supplementary Materials

Supplementary material are available online at www.mdpi.com/1099-4300/20/1/70/s1.

## Appendix A. Proof of Proposition 2

That G∩B⊆D follows from the fact that only dyadic rationals have a non-unique binary expansion. In particular, the preimage of x∈D consists of two elements, namely

(A1) $$b^{\prime }:=\left(b^{n}0000000\cdots \right)$$

and

(A2) $$b^{\prime \prime }:=\left(b^{n-1}{\bar{b}}_{n}1111111\cdots \right).$$

By the properties of polarization and with the assumption that 0<I(W)<1, we have that $0<I\left(W_{2^{n}}^{b^{n-1}{\bar{b}}_{n}}\right),I\left(W_{2^{n}}^{b^{n}}\right)<1$, and hence also $0<Z\left(W_{2^{n}}^{b^{n-1}{\bar{b}}_{n}}\right),Z\left(W_{2^{n}}^{b^{n}}\right)<1$. Moreover, it follows from Lemma 2 and (9) that

(A3) $$Z\left(W_{\infty }^{b^{\prime \prime }}\right)\leq \lim_{\ell \to \infty }{\left(Z(W_{2^{n}}^{b^{n-1}{\bar{b}}_{n}})\right)}^{2^{\ell }}=0$$

from which we obtain $I(W_{\infty }^{b^{\prime \prime }})=1$ and x∈G. Similarly, with Lemma 2 and (10) we obtain

(A4) $$Z\left(W_{\infty }^{b^{\prime }}\right)\geq \lim_{\ell \to \infty }\sqrt{1-{\left(1-Z^{2}\left(W_{2^{n}}^{b^{n}}\right)\right)}^{2^{\ell }}}=1.$$

Hence, $I(W_{\infty }^{b^{\prime }})=0$ and x∈B. Since this holds for every x∈D, we have that G∩B=D.

The proof that G\D and B\D are dense in [0,1] follows along similar lines. Specifically, we show that between every dyadic rational we can find rational numbers $x,x^{\prime }\in Q\backslash D$ such that x∈G and $x^{\prime }\in B$. To this end, fix $x_{1}=p/2^{n}$ and $x_{2}=\left(p+1\right)/2^{n}$. Let further $b^{n}$ be the terminating binary expansion of $x_{1}$, i.e., $f\left(b^{n}000\cdots \right)=x_{1}$.

Let $a^{k}$ be such that $a_{1}=\cdots =a_{k-1}=1$ and $a_{k}=0$. We can bound the polynomial $p_{a^{k}}$ from above:

$$p_{a^{k}}\left(z\right)=2z^{2^{k-1}}-z^{2^{k}}\leq 2z^{2^{k-1}}$$

The bound crosses *z* at z=0 and at $z^{*}=2^{-1/(2^{k-1}-1)}$, where $z^{*}$ can be made arbitrarily close to one for *k* sufficiently large. Now let $z_{i+1}=p_{a^{k}}\left(z_{i}\right)$, where $z_{0}=Z\left(W_{2^{n}}^{b_{n}}\right)$. It follows that $Z\left(W_{\infty }^{b^{n}a^{k}a^{k}a^{k}\cdots }\right)\leq \lim_{i\to \infty }z_{i}$. However, $z_{i}\to 0$ if $z_{0}<z^{*}$, hence if *k* is sufficiently large such that this holds, then $Z(W_{\infty }^{b^{n}a^{k}a^{k}a^{k}\cdots })\leq 0$. Thus, $I(W_{\infty }^{b^{n}a^{k}a^{k}a^{k}\cdots })=1$ and $x=f(b^{n}a^{k}a^{k}a^{k}\cdots )\in G$.

Recall that ${\bar{a}}^{k}$ is such that ${\bar{a}}_{1}=\cdots ={\bar{a}}_{k-1}=0$ and ${\bar{a}}_{k}=1$. We next bound the polynomial $q_{{\bar{a}}^{k}}$ from below:

(A5) $$q_{{\bar{a}}^{k}}\left(z\right)=1-{\left(1-z^{2}\right)}^{2^{k-1}}\geq 1-e^{-2^{k-1}z^{2}}$$

which intersects *z* at z=0, at some root $z^{*}$ that can be made arbitrarily close to zero for *k* sufficiently large, and at some root $z^{†}<1$ that tends to one if *k* becomes large. Note further that the slope of $q_{{\bar{a}}^{k}}$ equals $2^{k}z{\left(1-z^{2}\right)}^{2^{k-1}-1}$. By setting *k* sufficiently large, one can guarantee that this slope is smaller than one on the interval $\left[z^{†},1\right]$. Now let $z_{i+1}=q_{{\bar{a}}^{k}}\left(z_{i}\right)$, where $z_{0}=Z\left(W_{2^{n}}^{b_{n}}\right)$. Suppose further that we have chosen *k* sufficiently large such that $z_{0}>z^{*}$ and that the slope of $q_{{\bar{a}}^{k}}$ is smaller than one on the interval $\left[z^{†},1\right]$. We know that $q_{{\bar{a}}^{k}}\left(z\right)>z$ on the interval $\left(z^{*},z^{†}\right)$, since this is the case for the lower bound in (A5). However, since the slope of $q_{{\bar{a}}^{k}}$ is smaller than one on the interval $\left[z^{†},1\right]$ and since $q_{{\bar{a}}^{k}}$ intersects *z* at one, there can be no further intersection between $q_{{\bar{a}}^{k}}$ and *z* on the interval $\left[z^{†},1\right]$. Hence, $q_{{\bar{a}}^{k}}\left(z\right)>z$ on the interval $\left(z^{*},1\right]$, and $z_{i}\to 1$ since $z_{0}>z^{*}$. Since furthermore $Z\left(W_{\infty }^{b^{n}{\bar{a}}^{k}{\bar{a}}^{k}{\bar{a}}^{k}\cdots }\right)\geq \lim_{i\to \infty }z_{i}$, we obtain $Z(W_{\infty }^{b^{n}{\bar{a}}^{k}{\bar{a}}^{k}{\bar{a}}^{k}\cdots })\geq 1$. Thus, $I(W_{\infty }^{b^{n}{\bar{a}}^{k}{\bar{a}}^{k}{\bar{a}}^{k}\cdots })=0$ and $x^{\prime }=f\left(b^{n}{\bar{a}}^{k}{\bar{a}}^{k}{\bar{a}}^{k}\cdots \right)\in B$.

Since both $f(b^{n}a^{k}a^{k}a^{k}\cdots )$ and $f(b^{n}{\bar{a}}^{k}{\bar{a}}^{k}{\bar{a}}^{k}\cdots )$ are in the interval $\left(x_{1},x_{2}\right)$, this shows that between every two dyadic rationals, there are numbers *x* and $x^{\prime }$ that are good and bad. This proves that G\D and B\D are dense in [0,1].

## Appendix B. Proof of Proposition 3

**Lemma** **A1** ([25] (Lemma 11)). *For P-almost every realization b∈Ω, there exists a point θ(b)∈[0,1] such that*

(A6) $$\lim_{n\to \infty }p_{b^{n}}\left(z\right)=\left\{\begin{array}{cc} 0, & z\in [0,\theta (b))\\ 1, & z\in (\theta (b),1]. \end{array}\right.$$

Furthermore, the thus constructed RV θ is uniformly distributed on [0,1].

It can be easily verified that $g_{i}\left(1-z\right)=1-g_{1-i}\left(z\right)$ for i=0,1. Hence,

$$\begin{array}{cc} p_{b^{n}}\left(z\right) & =g_{b_{n}}\left(g_{b_{n-1}}\left(\cdots g_{b_{2}}\left(g_{b_{1}}\left(z\right)\right)\cdots \right)\right)\\ & =g_{b_{n}}\left(g_{b_{n-1}}\left(\cdots g_{b_{2}}\left(1-g_{{\bar{b}}_{1}}\left(1-z\right)\right)\cdots \right)\right)\\ & =g_{b_{n}}\left(g_{b_{n-1}}\left(\cdots 1-g_{{\bar{b}}_{2}}\left(g_{{\bar{b}}_{1}}\left(1-z\right)\right)\cdots \right)\right)\\ & =1-g_{{\bar{b}}_{n}}\left(g_{{\bar{b}}_{n-1}}\left(\cdots g_{{\bar{b}}_{2}}\left(g_{{\bar{b}}_{1}}\left(1-z\right)\right)\cdots \right)\right)\\ & =1-p_{{\bar{b}}^{n}}\left(1-z\right). \end{array}$$

Now suppose that $b\notin f^{-1}\left(D\right)$ and that θ(b) is defined. If 0≤z<θ(b), then 1−θ(b)<1−z≤1. Since, by Lemma A1, 0≤z<θ(b) implies $p_{b^{n}}\left(z\right)\to 0$ and $p_{{\bar{b}}^{n}}\left(1-z\right)\to 1$, we get $\theta \left(\bar{b}\right)=1-\theta \left(b\right)$. Now set ϑ(f(b)):=θ(b). Since $b+\bar{b}=11111\cdots$, it follows from the linearity of *f* that $f\left(b\right)+f\left(\bar{b}\right)=f\left(b+\bar{b}\right)=1$, i.e., if x∉D has binary expansion *b*, then 1−x has binary expansion $\bar{b}$. Therefore, for all x∉D for which $\theta (f^{-1}\left(x\right))$ is defined, we have ϑ(1−x)=1−ϑ(x).

Recall that, by Lemma 2, we have

(A7) $$Z\left(W_{2^{n}}^{b^{n}}\right)\leq p_{b^{n}}\left(Z\left(W\right)\right)=g_{b_{n}}\left(g_{b_{n-1}}\left(\cdots g_{b_{1}}\left(Z\left(W\right)\right)\cdots \right)\right).$$

If Z(W)<ϑ(x), then by Lemma A1, $Z\left(W_{\infty }^{b}\right)\leq \lim_{n\to \infty }p_{b^{n}}\left(Z\left(W\right)\right)=0$ and x∈G. If *W* is a BEC, then (A7) holds with equality. Thus, by Lemma A1, if Z(W)>ϑ(x), then $Z\left(W_{\infty }^{b}\right)=\lim_{n\to \infty }p_{b^{n}}\left(Z\left(W\right)\right)=1$ and x∈B.

## Appendix C. Proof of Proposition 4

For the proof we utilize properties of *f* derived in the proof of [26] (Theorem 2.1, p. 7). Specifically, let E⊂Ω be the set of all binary sequences with infinitely many zeros, i.e., *E* contains the binary expansion of all numbers x∈[0,1]\D and the terminating binary expansions of all numbers x∈D. Since Ω\E is countable, *E* is a Borel set. The function *f* restricted to *E*, f:E→[0,1], is bijective and measurable (since *f* is measurable by Lemma 1). Finally, the inverse function $f^{-1}:\;\left[0,1\right]\to E$ is measurable, i.e., for all A∈A, A⊆E, we have $f\left(A\right)\in B_{\left[0,1\right]}$.

The set *E* contains all binary sequences except for non-terminating expansions of dyadic rationals, which lead to good channels by the proof of Proposition 2. Thus, we have

(A8) $$\left\{b\in \Omega :\;I\left(W_{\infty }^{b}\right)=0\right\}\cap E=\left\{b\in \Omega :\;I\left(W_{\infty }^{b}\right)=0\right\}.$$

Since this set has probability 1−I(W) by Proposition 1, it is a Borel set (otherwise, it would not be measurable). However, since $f(\left\{b\in E:\;I\left(W_{\infty }^{b}\right)=0\right\})=B$, it follows that B is a Borel set of [0,1]. That G is a Borel set can be shown along similar lines, with *E* containing all binary sequences with infinitely many ones.

Every Borel set is Lebesgue measurable. To evaluate the Lebesgue measure of B, note that

(A9) $$f^{-1}\left(B\right)=\left\{b\in \Omega :\;I\left(W_{\infty }^{b}\right)=0\right\}\cup f^{-1}\left(D\right).$$

Since $B\in B_{\left[0,1\right]}$, we get from Lemma 1 that $\lambda \left(B\right)=P(f^{-1}\left(B\right))$. By the monotonicity and countable subadditivity of measures, we have

(A10) $$P\left(\left\{b\in \Omega :\;I\left(W_{\infty }^{b}\right)=0\right\}\right)\leq P\left(f^{-1}\left(B\right)\right)\leq P\left(\left\{b\in \Omega :\;I\left(W_{\infty }^{b}\right)=0\right\}\right)+\underset{=\lambda (D)=0}{\underset{︸}{P(f^{-1}\left(D\right))}}.$$

Hence, by Proposition 1, $\lambda \left(B\right)=P\left(\left\{b\in \Omega :\;I\left(W_{\infty }^{b}\right)=0\right\}\right)=1-I\left(W\right)$. The proof for the set of good channels follows along the same lines.

Since the one-dimensional Hausdorff measure of a Borel set equals its Lebesgue measure [1] (Equation (3.4), p. 45), it immediately follows that G and B have a Hausdorff dimension equal to one.

## Appendix D. Proof of Proposition 5

Since the dyadic rationals are self-similar and since D⊂G from Proposition 2, one has for all *n* and *k*,

(A11) $$G_{n}\left(k\right)\cap D=2\left(G_{n+1}\left(2k\right)\cap D\right)-k2^{-n}.$$

We now treat those values in [0,1] that are not dyadic rationals. If $b_{k}^{n}=\left(b_{1}b_{2}\cdots b_{n}\right)$ is the terminating binary expansion of $\left(k-1\right)2^{-n}$, every value in $\left[\left(k-1\right)2^{-n},k2^{-n}\right]$ has a binary expansion $b_{k}^{n}a$ for some a∈Ω, where $b_{n}=1$ if and only if (k−1) is odd. Similarly, and since (2k−1) is always odd, every value in $\left[\left(2k-1\right)2^{-n-1},k2^{-n}\right]$ has a binary expansion $b_{k}^{n}1a^{\prime }$ for some $a^{\prime }\in \Omega$. Assume that $a^{\prime }=a$. Then, by Lemmas 3 and 4, $W_{\infty }^{b_{k}^{n}a}≼W_{\infty }^{b_{k}^{n}1a}$ for all *a*. Hence, if $f\left(b_{k}^{n}a\right)\in G_{n}\left(k\right)$, then $f\left(b_{k}^{n}1a\right)\in G_{n+1}\left(2k\right)$. It remains to show that $2f\left(b_{k}^{n}1a\right)-f\left(b_{k+1}^{n}\right)=f\left(b_{k}^{n}a\right)$:

$$\begin{array}{cc} f\left(b_{k}^{n}a\right)+f\left(b_{k+1}^{n}\right) & =f\left(b_{k}^{n}\right)+2^{-n}f\left(a\right)+f\left(b_{k+1}^{n}\right)\\ & =\left(k-1\right)2^{-n}+2^{-n}f\left(a\right)+k2^{-n}\\ & =\left(2k-1\right)2^{-n}+2^{-n}f\left(a\right)\\ & =2\left(2k-1\right)2^{-n-1}+2\times 2^{-n-1}f\left(a\right)\\ & =2f\left(b_{k}^{n}1\right)+2\times 2^{-n-1}f\left(a\right)\\ & =2f(b_{k}^{n}1a) \end{array}$$

**Proof** **for Symmetric Channels.** Since (2k−2) is always even, every value in $\left[\left(2k-2\right)2^{-n-1},\left(2k-1\right)2^{-n-1}\right]$ has a binary expansion $b_{k}^{n}0a$ for some a∈Ω. Then, by Lemmas 3 and 4, $W_{\infty }^{b_{k}^{n}0a}≼W_{\infty }^{b_{k}^{n}a}$ for all *a*. Hence, if $f\left(b_{k}^{n}0a\right)\in G_{n+1}\left(2k\right)$, then $f\left(b_{k}^{n}a\right)\in G_{n}\left(k\right)$. It remains to show that $2f\left(b_{k}^{n}0a\right)-f\left(b_{k}^{n}\right)=f\left(b_{k}^{n}a\right)$:

$$\begin{array}{cc} f\left(b_{k}^{n}a\right)+f\left(b_{k}^{n}\right) & =f\left(b_{k}^{n}\right)+2^{-n}f\left(a\right)+f\left(b_{k}^{n}\right)\\ & =\left(k-1\right)2^{-n}+2^{-n}f\left(a\right)+\left(k-1\right)2^{-n}\\ & =\left(2k-2\right)2^{-n}+2^{-n}f\left(a\right)\\ & =2\left(2k-2\right)2^{-n-1}+2\times 2^{-n-1}f\left(a\right)\\ & =2f\left(b_{k}^{n}0\right)+2\times 2^{-n-1}f\left(a\right)\\ & =2f(b_{k}^{n}0a) \end{array}$$

☐

## Appendix E. Proof of Proposition 6

Note that in Definition 4 we can take the binary logarithm on both sides of the inequality to get the condition

(A12) $$x\in H\left(\rho \right)\Leftrightarrow \exists b\in f^{-1}\left(x\right):\;\underset{n\to \infty }{\lim\;\inf}\;w\left(b^{n}\right)-n\rho \geq 0.$$

We first show that D⊂H(ρ) for every ρ∈[0,1). To this end, consider the non-terminating expansion of x∈D, i.e., there is a $b^{k}\in {\left\{0,1\right\}}^{k}$ such that $f(b^{k}1111\cdots )=x$. Hence, $w(b^{n})\geq n-k$ for n≥k and, for ρ<1,

(A13) $$\begin{array}{cc} \underset{n\to \infty }{\lim\;\inf}\;w\left(b^{n}\right)-n\rho & \geq \lim_{n\to \infty }n\left(1-\rho \right)-k=\infty . \end{array}$$

We next show that also H(ρ)\D is dense in [0,1] for ρ<1. We do so by showing that between any two dyadic rationals $x_{1}=p/2^{n}$ and $x_{2}=\left(p+1\right)/2^{n}$, there exists a rational number x∈Q\D that is in H(ρ). Let $b^{\ell }$ be the terminating binary expansion of $x_{1}$, i.e., $f\left(b^{\ell }000\cdots \right)=x_{1}$. Furthermore, let $a^{k}$ be such that $a_{1}=\cdots =a_{k-1}=1$ and $a_{k}=0$. We set $b=b^{\ell }a^{k}a^{k}a^{k}\cdots$ and get $f\left(b\right)\in \left(x_{1},x_{2}\right)$. One can show that $w\left(b^{n}\right)\geq n-\ell -1-\frac{n-\ell }{k}$. Hence, choosing k>1/(1−ρ), ρ<1, leads to

(A14) $$\begin{array}{cc} \underset{n\to \infty }{\lim\;\inf}\;w\left(b^{n}\right)-n\rho & \geq \lim_{n\to \infty }n\left(1-\frac{1}{k}-\rho \right)-\ell -\frac{\ell }{k}-1=\infty . \end{array}$$

This proves that x∈H(ρ) if ρ<1, from which follows that H(ρ)\D is dense in [0,1].

We finally show that the there exists a non-dyadic rational number $x\in (x_{1},x_{2})$ such that x∉H(ρ). From this follows that also the complement of H(ρ)\D is dense in [0,1]. To this end, let ${\bar{a}}^{k}$ be such that ${\bar{a}}_{1}=\cdots ={\bar{a}}_{k-1}=0$ and ${\bar{a}}_{k}=1$. Set $b=b^{\ell }{\bar{a}}^{k}{\bar{a}}^{k}{\bar{a}}^{k}\cdots$. One can show that $w\left(b^{n}\right)\leq \frac{n}{k}+\ell -\frac{\ell }{k}+1$. Hence, choosing k>1/ρ, ρ>0, leads to

(A15) $$\begin{array}{cc} \underset{n\to \infty }{\lim\;\inf}\;w\left(b^{n}\right)-n\rho & \leq \lim_{n\to \infty }n\left(\frac{1}{k}-\rho \right)+\ell -\frac{\ell }{k}+1=-\infty . \end{array}$$

Therefore, x∉H(ρ) if ρ>0.

## Appendix F. Proof of Proposition 7

By Example 4, dyadic rationals are not simply normal, hence let N⊂[0,1]\D be the set of simply normal numbers in [0,1]. Note that *f* is bijective on N by Lemma 1. We furthermore have λ(N)=1, hence [0,1]\N is a Lebesgue null set (both N and its complement are measurable). Every subset of a Lebesgue null set is a Lebesgue null set and, a fortiori, Lebesgue measurable.

By Lemma 5 we have

(A16) $$\forall b\in f^{-1}\left(N\right):\;w\left(b^{n}\right)=\frac{1}{2}n+o\left(n\right).$$

Fix ρ. Then,

(A17) $$\underset{n\to \infty }{\lim\;\inf}\;w\left(b^{n}\right)-n\rho =\lim_{n\to \infty }n\left(\frac{1}{2}-\rho \right)+o\left(n\right).$$

If ρ<1/2, then this limit diverges to infinity, and hence N⊂H(ρ). Thus, [0,1]\H(ρ) is a subset of [0,1]\N, hence measurable, from which measurability of H(ρ) follows. Since λ(N)=1, we have λ(H(ρ))=1. If ρ>1/2, the limit diverges to minus infinity, and hence N¬⊂H(ρ). Thus, H(ρ)⊂[0,1]\N, from which λ(H(ρ))=0 follows.

Now let ρ=1/2. We define a random variable *B* on our probability space, such that for all b∈Ω, B(b)=b. *B* is a sequence of independent, identically distributed Bernoulli-1/2 random variables, i.e., for all *i* we have $P\left(B_{i}=1\right)=P\left(B_{i}=0\right)=1/2$. We now evaluate

(A18) $$P\left(\underset{n\to \infty }{\lim\;\inf}\;w\left(B^{n}\right)-\frac{n}{2}\geq 0\right).$$

Consider the simple random walk $S_{n}:=w\left(B^{n}\right)-\frac{n}{2}$. Let $N_{0}\left(n\right)$ be the number of zero crossings of the sequence $S_{1},\ldots ,S_{n}$ and let $N_{0}\left(n,b\right)$ be the number of zero crossings corresponding to the realization b∈Ω. The event ${\lim\;\inf}_{n\to \infty }w\left(b^{n}\right)-\frac{n}{2}\geq 0$ can only happen if the realization of $S_{n}$ corresponding to *b* has only finitely many zero crossings, i.e.,

$$\begin{array}{cc} \left\{b\in \Omega :\;\underset{n\to \infty }{\lim\;\inf}\;w\left(b^{n}\right)-\frac{n}{2}\geq 0\right\} & \subseteq \{b\in \Omega :\;\exists R\in N_{0}:\lim_{n\to \infty }N_{0}\left(n,b\right)\leq R\}\\ & =\overset{\infty }{\underset{R=0}{⋃}}\left\{b\in \Omega :\;\lim_{n\to \infty }N_{0}\left(n,b\right)\leq R\right\}\\ & =\overset{\infty }{\underset{R=0}{⋃}}\underset{n\to \infty }{\lim\;\inf}\left\{b\in \Omega :\;N_{0}\left(n,b\right)\leq R\right\} \end{array}$$

and hence

(A19) $$\begin{array}{c} P\left(\{b\in \Omega :\;\underset{n\to \infty }{\lim\;\inf}\;w\left(b^{n}\right)-\frac{n}{2}\geq 0\}\right)\\ \leq \sum_{R=0}^{\infty }P\left(\underset{n\to \infty }{\lim\;\inf}\left\{b\in \Omega :\;N_{0}\left(n,b\right)\leq R\right\}\right)\\ \leq \sum_{R=0}^{\infty }\lim_{n\to \infty }P\left(N_{0}\left(n\right)\leq R\right) \end{array}$$

where the second inequality is due to Fatou’s lemma [27] (Lemma 1.28, p. 23).

With [28] (Chapter III.5, p. 84)

(A20) $$P\left(N_{0}\left(n\right)=R\right)=2P\left(S_{2n+1}=2R+1\right)$$

we get

P(N0(n)≤R)=2∑r=0RP(S2n+1=2r+1)=(a)2∑r=0R2n+1n−r2−2n−1≤2−2n∑r=0R2n+2n+1=2−2n(R+1)2n+2n+1≤(b)2−2n(R+1)e22n+21(n+1)π=4e(R+1)(n+1)π

where (a) is [28] (Equation (2.2), p. 75) and (b) is due to Stirling’s approximation [29] (Equation (6.1.38), p. 257). Since this probability tends to zero as n→∞, we have by (A19)

(A21) $$P\left(\underset{n\to \infty }{\lim\;\inf}\;w\left(B^{n}\right)-\frac{n}{2}\geq 0\right)=0.$$

Since the set inside the probability measure is thus measurable, we can apply the same reasoning as in the proof of Proposition 4 to argue that H(1/2) is Lebesgue measurable. We then obtain λ(H(1/2))=0 which completes the proof for the Lebesgue measure.

We now turn to the proof of the Hausdorff dimension. For ρ<1/2, H(ρ) has full Lebesgue measure. Since every Lebesgue measurable set has a Borel subset with the same Lebesgue measure, and since Hausdorff dimension is monotonic, we have d(H(ρ))=1 for ρ<1/2.

For ρ≥1/2, we define

(A22) $${\tilde{N}}_{\xi }:=\left\{x\in \left[0,1\right]:\;\exists b\in f^{-1}\left(x\right):\lim_{n\to \infty }\frac{w(b^{n})}{n}=\xi \right\}$$

for some ξ∈(0,1). Note that ${\tilde{N}}_{1/2}=N$. By [30] (cf. [21] (Chapter 8) for further notes), the Hausdorff dimension of this set is given by (Interestingly, in Eggleston’s paper, the dimension was not connected to entropy; it was submitted earlier in the same year as Shannon’s Mathematical Theory of Communication was published).

(A23) $$d\left({\tilde{N}}_{p}\right)=h_{2}\left(\xi \right).$$

Reasoning as in the proof for the Lebesgue measure, ${\tilde{N}}_{\xi }\subset H\left(\rho \right)$ if ξ>ρ and ${\tilde{N}}_{p}⊄H\left(\rho \right)$ if ξ<ρ. As a consequence,

(A24) $$\overset{\infty }{\underset{n=1}{⋃}}{\tilde{N}}_{\rho +1/n}\subset H\left(\rho \right).$$

For a countable sequence of sets $A_{n}$, Hausdorff dimension satisfies [1] (p. 49)

(A25) $$d\left(\overset{\infty }{\underset{n=1}{⋃}}A_{n}\right)=\underset{n\geq 1}{\sup}d\left(A_{n}\right)$$

and hence, by the monotonicity of Hausdorff dimension [1] (p. 48),

(A26) $$d\left(H\left(\rho \right)\right)\geq \underset{n\geq 1}{\sup}h_{2}\left(\rho +1/n\right)=h_{2}\left(\rho \right)$$

where the last equality follows from the fact that the binary entropy function decreases with increasing ρ for ρ≥1/2. In particular, for ρ=1/2, d(H(ρ))=1. This completes the proof.

## Appendix G. Proof of Proposition 8

The proof follows along the lines of the proof of Proposition 5. Let again $b_{k}^{n}$ be the terminating expansion of $\left(k-1\right)2^{-n}$ and let a∈Ω. The connections between the sequences $b:=b_{k}^{n}a$, $b_{-}:=b_{k}^{n}0a$, and $b_{+}:=b_{k}^{n}1a$ have been established above. To prove the theorem, we have to show that

(A27) $$\begin{array}{c} \underset{m\to \infty }{\lim\;\inf}\;w\left(b_{-}^{m}\right)-\rho m\geq 0 \end{array}$$

(A28) $$\begin{array}{c} \Rightarrow \underset{m\to \infty }{\lim\;\inf}\;w\left(b^{m}\right)-\rho m\geq 0 \end{array}$$

(A29) $$\begin{array}{c} \Rightarrow \underset{m\to \infty }{\lim\;\inf}\;w\left(b_{+}^{m}\right)-\rho m\geq 0. \end{array}$$

This is obtained by

(A30) $$\begin{array}{cc} \underset{m\to \infty }{\lim\;\inf}\;w\left(b_{-}^{m}\right)-\rho m & =w\left(b_{k}^{n}0\right)-\rho \left(n+1\right)+\underset{m\to \infty }{\lim\;\inf}\;w\left(a^{m}\right)-\rho m\\ & =w\left(b_{k}^{n}\right)-\rho n-\rho +\underset{m\to \infty }{\lim\;\inf}\;w\left(a^{m}\right)-\rho m\\ & \leq w\left(b_{k}^{n}\right)-\rho n+\underset{m\to \infty }{\lim\;\inf}\;w\left(a^{m}\right)-\rho m\\ & \leq w\left(b_{k}^{n}\right)-\rho n+\left(1-\rho \right)+\underset{m\to \infty }{\lim\;\inf}\;w\left(a^{m}\right)-\rho m \end{array}$$

(A31) $$\begin{array}{c} \;=w\left(b_{k}^{n}1\right)-\rho \left(n+1\right)+\underset{m\to \infty }{\lim\;\inf}\;w\left(a^{m}\right)-\rho m \end{array}$$

where (A30) equals (A28) and where (A31) equals (A29). The inequalities yield the desired result.
